# Influence of Foot and Legwear Color on Lower-Limb Temperature in Baseball Players Under Heat Stress

**DOI:** 10.3390/sports13100369

**Published:** 2025-10-21

**Authors:** Manato Seguchi, Yoko Iio, Saimi Yamamoto, Tsukasa Yamamoto, Harumi Ejiri, Yuka Aoyama, Morihiro Ito

**Affiliations:** 1 Graduate School of Life and Health Sciences, Chubu University, 1200 Matsumoto-cho, Kasugai-shi 487-8501, Aichi, Japan; 2 Support for Pioneering Research Initiated by the Next Generation (SPRING), Chubu University, 1200 Matsumoto-cho, Kasugai-shi 487-8501, Aichi, Japan; 3 Department of Lifelong Sports and Health Sciences, College of Life and Health Sciences, Chubu University, 1200 Matsumoto-cho, Kasugai-shi 487-8501, Aichi, Japan; 4 Department of Early Childhood Education, College of Contemporary Education, Chubu University, 1200 Matsumoto-cho, Kasugai-shi 487-8501, Aichi, Japan; 5 Center for Nursing Practicum Support, Chubu University, 1200 Matsumoto-cho, Kasugai-shi 487-8501, Aichi, Japan; 6 Department of Nursing, College of Life and Health Science, Chubu University, 1200 Matsumoto-cho, Kasugai-shi 487-8501, Aichi, Japan; 7 Department of Clinical Engineering, College of Life and Health Sciences, Chubu University, 1200 Matsumoto-cho, Kasugai-shi 487-8501, Aichi, Japan; 8 Department of Biomedical Sciences, College of Life and Health Science, Chubu University, 1200 Matsumoto-cho, Kasugai-shi 487-8501, Aichi, Japan

**Keywords:** baseball, foot and legwear, heat stress, heat stroke, lower-limb temperature, lower-limb temperature, low-temperature burns, under the blazing heat

## Abstract

**Background:** Elevated global temperatures increase the risk of heat-stroke among athletes exercising in hot conditions. Japanese high school baseball tournaments occur during peak summer, raising concerns regarding heat-related health issues. We examined whether the color of footwear and legwear affects lower-limb temperature, exploring approaches to prevent heat-related health problems. **Methods:** Eight mannequin legs were fitted with shoes, socks, and baseball stirrup socks in white or black combinations. Plantar and shin surface temperatures were recorded for 120 min on both dirt and artificial turf at wet-bulb globe temperatures above 30 °C and compared across color combinations. Reflectance spectra of shin legwear were also measured. **Results:** Plantar and shin surface temperatures increased under all conditions. On the dirt field, mannequins wearing all-black gear (shoe, sock, and baseball stirrup sock) exhibited plantar temperatures exceeding 45 °C and shin temperatures over 50 °C. The highest shin temperature occurred with the white shoe/black baseball stirrup sock combination. Temperature increases were smaller for all-white items compared with all-black items. Reflectance spectra showed that white baseball stirrup socks strongly reflected both visible and infrared light. **Conclusions:** Footwear and legwear color significantly influence lower-limb temperature increases during baseball games in summer heat, especially when wearing all-black items. White gear may help prevent heat-related health problems and improve performance in baseball and other outdoor sports.

## 1. Introduction

The average global temperature has increased by approximately 1 °C since the 20th century. Additionally, the global average surface temperature in March 2023 was reported as the second highest for that month since records began in 1850. Thus, the impact of rising environmental temperatures on health has gained attention and become a global issue [1]. In hot environments, the human body experiences excessive heat transfer, increased metabolic heat production due to strenuous physical labor, and dehydration. These factors can reduce the body’s ability to manage heat stress, decrease heat dissipation capacity, and increase core body temperature, adversely affecting the muscular, cardiovascular, central nervous, and renal systems [2,3,4,5]. Exposure to hot environments increases physiological and perceptual responses, leading to heightened heat stress; reduced safety, health, and work capacity; and an elevated risk of heat stroke [2,4,5,6].

Thermoregulation during exercise in hot environments is primarily achieved through sweat evaporation, with the body balancing heat production and release [7]. Thermoregulation is especially important for athletes, who often compete in hot and humid conditions where thermoregulation capacity is reduced, and any disruption increases heat-stroke risk and adversely affects performance [8,9]. Athletes utilize various cooling strategies, including cooling or ice vests, neck coolers, cold drinks, and ice slurries, to maintain thermoregulation during training [8]. However, using these strategies during exercise can be difficult due to physical limitations, such as excess weight from wearing multiple products, skin inflammation and discomfort caused by friction with wearing products [10], and intake issues [11]. Furthermore, rules and regulations often prohibit athletes from using these cooling options.

Conversely, sportswear made from breathable, lightweight synthetic materials is designed to reduce heat-insulating effects by improving moisture transfer (sweat absorption), promoting evaporative heat loss, maximizing wearer comfort, and ultimately improving performance [12,13]. Sportswear selection supports thermoregulation during exercise in hot environments and contributes to comfort through various sensory inputs, including psychological, sensory, and physical movement factors [14].

In this study, we focused on baseball, a popular sport played by people of all ages, genders, and physical abilities worldwide, although cultural differences exist across regions. In Japan, amateur baseball has a longer history as a popular sport than professional baseball. In particular, student baseball has become a summer tradition, with the national high school tournament held during the hottest time of the year. Currently, sports activities, including baseball, in extreme heat are recognized as a major health concern for athletes, and prevention and targeting of heat-related health disorders is extremely important [15,16].

We hypothesized that appropriate color selection for outdoor sportswear reduces lower-limb temperature rises in summer, with the understanding that clothing color selection is an important factor in reducing heat stress while exercising under intense heat. This offers an important adaptation strategy for climate change. Although the influence of clothing color on temperature changes and heat stress is typically studied in home economics and clothing science, these academic fields have shown little interest in sportswear, leaving limited data available [17].

Therefore, in this work, we aimed to investigate lower-limb temperature changes based on the color of sportswear—white or black. Among sportswear products, we focused on three items worn on the feet and legs: shoes, socks, and baseball stockings, and used mannequin models to compare and examine changes in lower-limb temperature during baseball games played under direct sunlight based on actual measured data.

## 2. Materials and Methods

### 2.1. Environmental Conditions

The experiment was conducted between 10:00 and 15:00 in August 2024 at two locations (a dirt field in a baseball field and an artificial turf in an all-weather stadium) in Ka-sugai City, Aichi Prefecture (E 136°58′19.8″ N 35°14′51.3″). Using a heat-stroke index data logger (#AD-5695DLB, A&D Company, Tokyo, Japan), measurements were taken continuously during the experiment. The mean temperature during measurement was 37.8 ± 1.42 °C, while the mean humidity was 43.8 ± 1.91%. The experiment was conducted with a wet-bulb globe temperature (WBGT) of ≥30 °C and continuous exposure to direct sunlight.

### 2.2. Foot and Legwear Items

**Figure 1:**
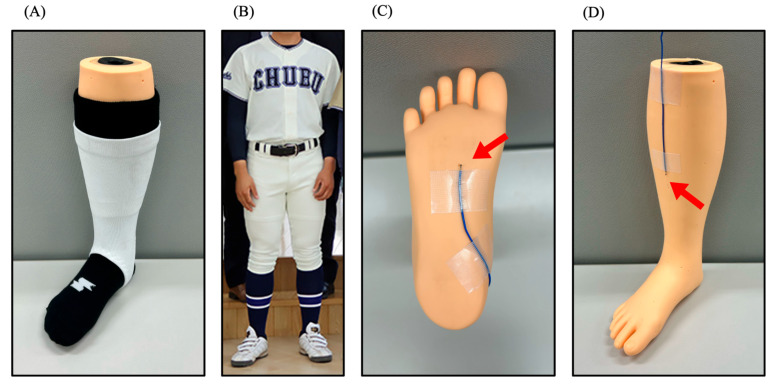
Style of wearing foot and legwear items and positions of temperature measurement: (**A**) A knee-length black sock was attached to the mannequin, with a white baseball stirrup sock worn over it. Therefore, the surface of the mannequin was covered by both the black sock and white baseball stirrup sock. (**B**) The legwear-wearing style in this study shows stirrup socks exposed, as stipulated in student baseball in Japan. (**C**) A temperature sensor was placed on the plantar surface of the mannequin, with the red arrow indicating the temperature sensor. (**D**) A temperature sensor was placed on the anterior shin surface of the mannequin, with the red arrow indicating the temperature sensor.

Baseball socks are typically knee-length, and knee-length baseball stirrup socks are commonly worn over them (Figure 1A). There are two main wearing styles: in one style, the hems of the uniform are folded in below the knees, with the lower legs covered with socks and baseball stirrup socks, leaving the baseball stirrup socks exposed (Figure 1B), and in the other style, the lower legs are covered with socks, baseball stirrup socks, and the uniform, with the stirrup socks unexposed. The first style was used in this study, as stipulated in student baseball in Japan.

Eight mannequin legs (below the knee to the toes) were used, each fitted in standard baseball gear—shoes, socks, and baseball stirrup socks—in either white or black. The items were worn in eight different color combinations. Shoes from Puma (Anzarun Lite, Puma White-Puma White; #371128 03. Puma Black-Puma Black; #371128 01, PUMA SE, Herzogenaurach, Germany), socks from SSK Corporation (Baseball White Socks; #YA2134 10. Baseball Black Socks; # YA2134 90, SSK CORPORATION, Osaka, Japan), and baseball stirrup socks from ZETT Corporation (Ultra-low-cut stockings White; #BK85A 1100. Ultra-low-cut stockings Black; #BK85A 1900, ZETT CORPORATION, Osaka, Japan) were used.

### 2.3. Temperature Measurements

The temperatures of the plantar and anterior shin surfaces were measured by attaching temperature sensors of Thermocouple Thermometers (#HT-9815, Dongguan Xintai Instrument Co., Ltd., Dongguan, China) at identical positions on all mannequins before the mannequins wore foot and legwear items (Figure 1C,D). All sensors were calibrated in boiling water and ice water to verify accuracy, and none showed deviations. Sensors for the plantar surface were placed under the socks, while sensors for the anterior tibial surface were placed under the socks and baseball stirrup socks to record surface temperatures.

For measuring the temperature of the plantar surface, we prepared four combinations: two combinations of white and black shoes, and two combinations of white and black socks. Since there are no baseball stirrup socks on the soles of the feet (refer to Figure 1A), these were the four combinations examined. To evaluate four different combinations in a single session, four mannequins, each wearing one of the four different combination, were simultaneously placed at the test location for measurement. For shin surfaces temperature measurements, we prepared eight combinations: two combinations of white and black shoes, two combinations of white and black socks, and two combinations of white and black baseball stirrup socks. Eight mannequins wearing different combinations of shoes, socks, and baseball stirrup socks were placed simultaneously in the test area and measured. The shin surfaces temperature measurements involved evaluating each of the eight different combinations in one session.

Temperature measurements were recorded while the mannequins remained stationary for 120 min: every minute for the first 10 min, every 10 min from 10 to 60 min, and every 20 min from the 60th min onward. This procedure constituted one session; three sessions were conducted on both the dirt and artificial turf fields (six sessions total), all on separate days.

The results were averaged for each field based on the values obtained from three measurements (*n* = 3 sessions) under each wearing condition, and presented as the mean ± standard deviation. To assess changes in temperature with different color combinations, the difference between the mean temperature at the start of the measurement and the mean maximum temperature reached during the measurement was calculated.

### 2.4. Measurements of Reflectance Spectra

Reflectance spectra were measured using a portable spectroradiometer (#MS-720, EKO Instruments Co., Tokyo, Japan). Eight mannequins wearing shoes, socks, and baseball stockings were placed in the test field, and three independent experiments were conducted on both a dirt field and artificial turf. Measurements were taken over a wavelength range of 350–1050 nm, recording the spectrum of light reflected from baseball stirrup socks illuminated perpendicularly from the front. The sensor was positioned 10 cm from the measurement area using an attachment with a 45° aperture angle. Data collection and analysis were performed using the instrument’s supplied software. The data is one of the three results obtained from experiments conducted on each field type, presented here as a typical example (or a representative instance).

### 2.5. Human and Animal Rights

Ethical approval and informed consent were not required for this study as it did not involve human subjects.

### 2.6. Statistical Analysis

Statistical analysis was performed using SPSS, version 29 (IBM Corp., Armonk, NY, USA). We performed a Levene’s test, but since the assumption of equal variance was not satisfied, we used Welch’s *t*-test and Cohen’s *d* for effect size. The temperature differences for each condition obtained in Section 2.3, which consisted of *n* = 3 samples, were compared between the control combination (white shoes, white socks, and white baseball socks) and other color combinations in each field. The statistical significance level was set at *p* < 0.05. For the magnitude of Cohen’s *d* effect size, values of 0.2, 0.5, and 0.8 were set to represent small, medium, and large effects, respectively.

## 3. Results

### 3.1. Effect of the Color of Shoes and Socks on Plantar Temperature

**Figure 2:**
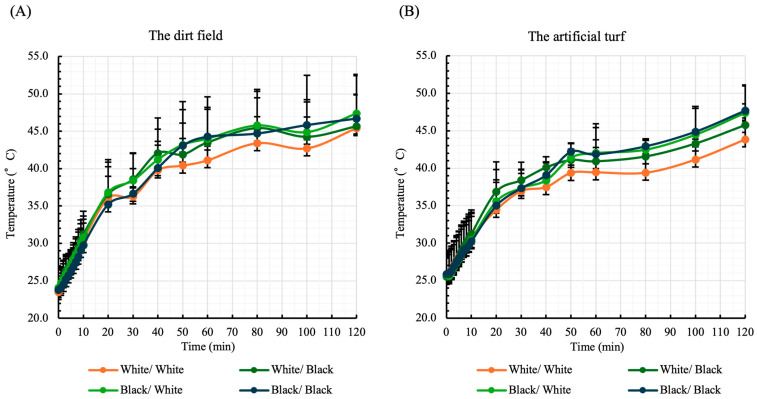
Temperature on the plantar surface. Measurements were taken by a temperature sensor attached to the plantar surface of mannequins for 120 min under the blazing heat. As indicated in the Methods, the measurements were taken on different days on the dirt field (**A**) and the artificial turf (**B**). The time-course results are expressed as means ± standard deviations. The colors of foot and legwear items are shown in the order of shoe/sock.

Figure 2 shows the temperatures measured on the plantar surface of the mannequins wearing shoes and socks in different color combinations. For all color combinations, the plantar surface temperature continued to increase from the start to the end of the measurement on both the dirt field and artificial turf. The maximum plantar surface temperature of the mannequin wearing a black shoe and a black sock on the dirt field reached 46.7 ± 5.73 °C (Figure 2A). Meanwhile, the maximum sole surface temperature of the mannequin wearing white shoes and white socks on the dirt field was 45.4 ± 4.55 °C (Figure 2A). On the artificial turf, the maximum sole surface temperature of the mannequin wearing black shoes and black socks was 47.7 ± 3.29 °C (Figure 2B). In contrast, the maximum foot sole surface temperature for the mannequin wearing white shoes and white socks on artificial turf was 43.9 ± 2.43 °C (Figure 2B).

### 3.2. Effect of Foot and Legwear Color on Shin Temperature

**Figure 3:**
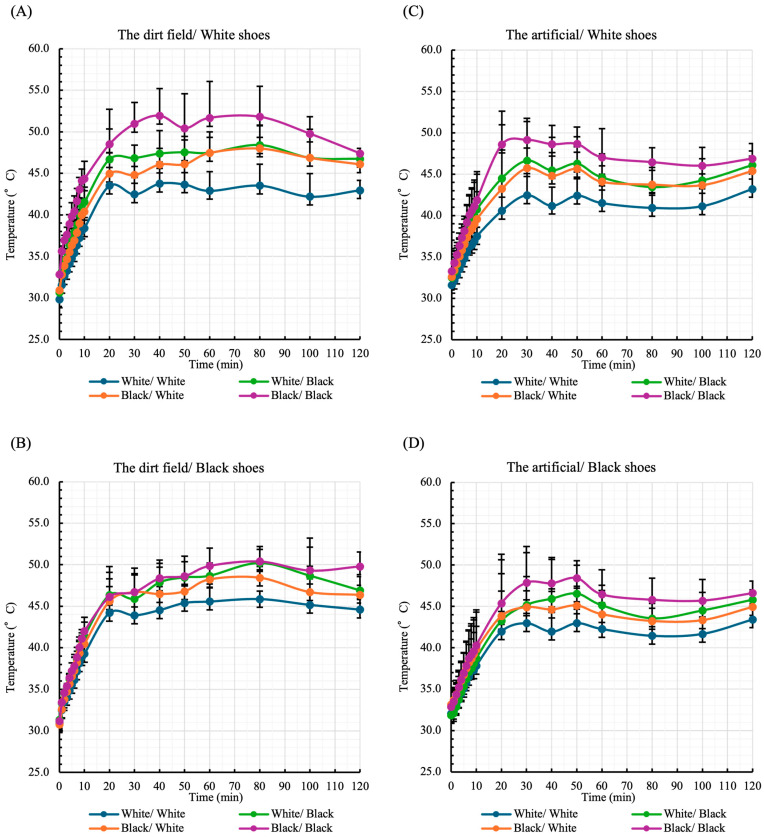
Temperature on the shin surface. Measurements were taken by a temperature sensor attached to the anterior shin surface of mannequins for 120 min under the blazing heat. As indicated in the Methods, the measurements were conducted on different days for the dirt field (**A**,**B**) and the artificial turf (**C**,**D**). The time-course results obtained in mannequins wearing a white shoe (**A**,**C**) and a black shoe (**B**,**D**) are expressed as means ± standard deviations. The colors of foot and legwear items are shown in the order of sock/baseball stirrup sock.

On a dirt field, the maximum shin temperature of a mannequin wearing white shoes was 43.8 ± 1.81 °C with white socks and white baseball stockings, and 51.9 ± 3.27 °C with black socks and black baseball stockings (Figure 3A). For mannequins wearing black shoes on the same field, the maximum shin temperature was 45.9 ± 0.97 °C with white socks and white baseball socks, and 50.4 ± 1.81 °C with black socks and black baseball socks (Figure 3B).

On artificial turf, the maximum shin temperature of mannequins wearing white shoes was 43.2 ± 1.85 °C with white socks and white baseball socks, and 49.2 ± 6.22 °C with black socks and black baseball socks (Figure 3C). For mannequins wearing black shoes on artificial turf, the maximum shin temperature was 43.4 ± 1.87 °C with white socks and white baseball socks, and 48.4 ± 2.11 °C with black socks and black baseball socks (Figure 3D).

### 3.3. Increase in Plantar Surface Temperature

Table 1 presents the difference between the mean temperature at the start of measurement and the mean maximum temperature reached during the measurement. On both the dirt field and artificial turf, none of the color combinations showed a statistically significant difference compared with the white shoe–white sock combination. On the dirt field, the temperature difference for the mannequin wearing a white shoe and white sock was 1.4 ± 7.46 °C greater than that for the mannequin wearing a white sock and black shoe. On artificial turf, the difference under the same combination was 3.6 ± 7.83 °C. Additionally, the difference for the white shoe–white sock combination was 2.2 ± 7.59 °C smaller than that for the white shoe–black sock combination.

### 3.4. Increase in Shin Surface Temperature

Table 2 presents the difference between the mean temperature at the start of measurement and the mean maximum temperature reached during the measurement. On dirt fields, the combination of white shoes, white socks, and white baseball stockings showed a significantly smaller temperature increase compared to the combinations of black shoes with white socks and black baseball stockings, or black shoes with black socks. On artificial turf, no significant differences were observed. Nevertheless, the combination of white shoes, white socks, and white baseball socks was 5.2 ± 3.54 °C cooler than the combination of white shoes, black socks, and black baseball socks, and 5.3 ± 1.67 °C cooler than the combination of black shoes, black socks, and black baseball socks.

### 3.5. Effect of the Light Reflected by Legwear

**Figure 4:**
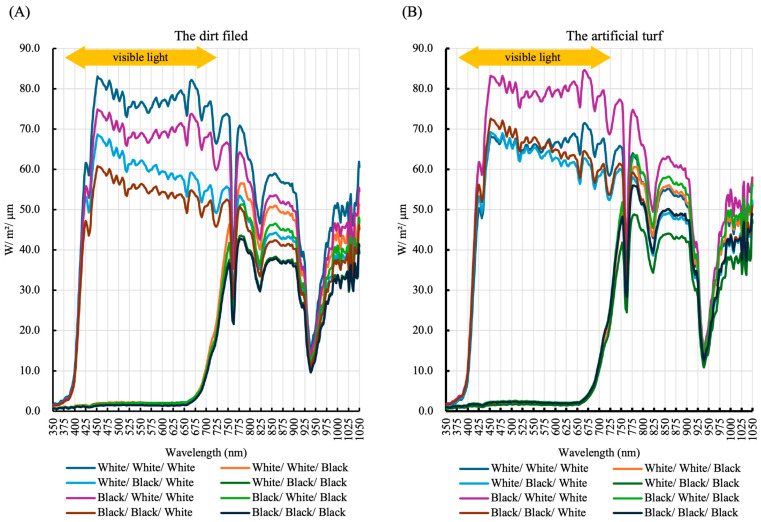
Reflectance spectrum for different color combinations. Light reflected from the anterior shin was measured in mannequins wearing various color combinations of shoes, socks, and baseball stirrup socks. Yellow arrows indicate the visible light range. Data represent one of three results obtained from experiments conducted on the dirt field (**A**) and artificial turf (**B**), respectively. Foot and legwear colors are presented in the order: shoe/sock/baseball stirrup sock.

Figure 4 presents the measurement results of natural light reflected by legwear. Mannequins wearing a white baseball stirrup socks reflected light across a wide range, including visible wavelengths. In contrast, mannequins wearing black baseball stirrup socks absorbed most of the light in the visible range. In the infrared range (wavelength ≥ 750 nm), black baseball stirrup socks absorbed more light than white baseball stirrup socks.

## 4. Discussion

In the present study, we experimentally examined the effect of different color combinations of baseball foot and legwear on the temperatures of the plantar and anterior shin surfaces of mannequins in a summer environment. We referred to previous studies that examined temperature changes using mannequins [18,19]. As shown in Figure 2, plantar temperature continued to increase from the start to the end of measurement, and under intense heat, the temperature tended to rise even beyond 120 min. In particular, plantar temperature was higher in mannequins wearing black shoes and black socks. Conversely, mannequins wearing white shoes and white socks exhibited lower plantar surface temperatures than those wearing shoes and socks in any other color combinations.

Additionally, compared with the combination of white shoes and white socks, none of the other combinations showed a significant difference in the increase from the start of measurement to the maximum temperature (Table 1). However, mannequins wearing black shoes exhibited a greater increase in plantar temperature than those wearing white shoes, even with the same sock color. These findings indicate that the color combination of baseball shoes and socks influences the increase in plantar surface temperature under extreme heat.

Although this study was conducted using mannequins, research on human participants is warranted. Human studies have shown that increases in lower-limb temperature are accompanied by rises in core temperature [20,21]. Furthermore, in the medical field, interventions to warm the soles of the feet have been shown to help maintain core temperature during surgery [22]. These findings suggest that humans may experience greater heat stress due to factors, such as body temperature, shoe humidity, and other physiological variables.

The black color generally has a high light absorption rate, likely due to the conversion of absorbed light energy into thermal energy. Tanaka [23] reported that black clothing absorbs heat radiation more easily, consistent with our findings. Furthermore, Ichinose et al. [17] showed that under direct sunlight, the surface temperature of black clothing was significantly higher than that of white clothing, and Tsuji et al. [24] highlighted the importance of wearing white sportswear when exercising outdoors in hot environments with a WBGT exceeding 28 °C. These findings align with our results, which showed high plantar temperatures in mannequins wearing black shoes and black socks.

As shown in Figure 3, shin surface temperature peaked approximately 30 min after the start of measurement and then remained constant or decreased. Regardless of study conditions, the highest shin temperature was observed in mannequins wearing black socks and black baseball stirrup socks. Interestingly, shin temperature was higher in mannequins wearing white shoes than in those wearing black shoes, with the maximum temperature on the dirt field exceeding the mean temperature by 14.0 ± 3.95 °C. Conversely, the lowest maximum shin temperature was observed in mannequins wearing white socks and white baseball stirrup socks, regardless of measurement location or shoe color. At both measurement locations, shin temperature was lower in mannequins wearing white shoes than in those wearing black shoes. These findings strongly suggest that shin temperature is influenced by the color combination of shoes, socks, and baseball stirrup socks.

Table 2 shows that the combination of white shoes, white socks, and white baseball stirrup socks, which had the lowest shin surface temperature on the dirt field (Figure 3), was associated with a significantly smaller increase in shin temperature compared with other combinations, except for black shoes with white socks, suggesting that this color combination prevented lower-limb temperature increases. Naito et al. reported that the absorption rate of solar radiation varies considerably depending on shirt color, with the surface temperature of a white shirt differing from that of a black shirt by >15 °C. Similarly, Nielsen [25] showed that black clothing absorbs sunlight more effectively, raising skin surface temperature. These findings suggest that the temperature increase observed with the socks in the present study likely involved sunlight absorption by black baseball stirrup socks, which increased mannequin temperatures.

Descriptive analysis revealed that, under the same legwear conditions, the plantar and tibial surface temperatures tended to be higher on the dirt field than on the artificial turf field. This is based on a comparison of the mean temperature values. This may be owing to differences in the thermal conductivity and insulation properties of the mannequin materials, the light-reflection characteristics of each field, and environmental ventilation. In addition, the temperature at the start of measurement was lower on the plantar surface than that on the shin surface. However, at 120 min, the temperature of the plantar surface was equal to or higher than that of the shin surface. This marked temperature increase on the plantar surface is likely because the shoes covered the entire feet, preventing the release of the heat accumulated in the soles, and because the heat released from the ground affected the soles.

On the dirt field, the temperature of the plantar surface exceeded 43.4 ± 4.69 °C at 60 min at the earliest and continued to increase until the end of measurement. Additionally, the temperature of the shin surface exceeded 48.5 ± 4.23 °C as early as 20 min into measurement and increased to 51.9 ± 3.27 °C at 40 min. Prolonged skin contact with a heat source above 44 °C can cause low-temperature burns, and the severity of these burns has been reported to increase with both exposure to temperatures between 44 °C and 51 °C and the duration of contact. Moreover, a human study revealed that plantar temperatures inside shoes exceeded 50 °C during daytime running in summer [26]. This strongly suggests that playing baseball under intense heat can be sufficient to cause low-temperature burns, depending on the color of clothing worn. Early-stage symptoms of low-temperature burns are mild and often not very painful, making them difficult to notice. These burns require particular attention, as they have been reported to frequently result in second- or third-degree lesions [27]. Therefore, clothing color selection is important when playing baseball in blazing heat.

Human legs play a substantial role in regulating core body temperature, serving as important sites of heat exchange for thermoregulation, in addition to supporting standing, walking, and locomotion. The soles and palms contain a high density of arteriovenous anastomoses, which are crucial for thermoregulation; they not only function as major sites of heat exchange but also influence core body temperature [28]. Taylor et al. [29] showed that heat dissipation occurs from the soles of the feet, with both sweat production and evaporative heat loss increasing during exercise or in hot environments. However, under conditions of high humidity and ambient temperature, sweat evaporation is insufficient, hindering cooling and exacerbating heat stress, which can lead to hyperthermia (heat stroke) [30]. The inside of shoes is prone to accumulating heat due to ambient temperature, humidity, solar radiation (direct and indirect), thermal radiation from the ground, and other factors. In the present study, plantar surface temperatures were at least 7.8 °C higher than the ambient temperature and continued to rise during measurement. This increase strongly indicates an elevated risk of heat-stress-related health problems. However, as measurements were taken with mannequins in a fixed position, dynamic conditions could not be simulated, and blood circulation in the lower limbs during movement could not be assessed.

Our findings suggest that white clothing worn on the lower limbs may suppress increases in lower-limb temperature compared to black clothing, potentially contributing not only to improved athletic performance but also to reduced health risks associated with heat stress. As shown in Figure 4, all mannequins with high reflection of visible light wore a white baseball stirrup sock, consistent with the color combinations that showed smaller increases in shin surface temperature. Especially, the results suggest that white clothing reflects a wide range of light wavelengths, reducing heat absorption. In fact, mannequins wearing a white sock and a white baseball stirrup sock on the dirt field exhibited greater light reflection when paired with a white shoe than with a black shoe (Figure 4), and the shin surface temperature was higher in mannequins wearing a white shoe than in those wearing a black shoe (Figure 3). This may be because some of the light reflected by the shoe was directed toward the shin. These findings strongly suggest that preventing heat-related health problems requires considering the color combinations of shoes, socks, and baseball stirrup socks, rather than simply changing shoe color from black to white—a key strength of this study.

This study has certain limitations. First, ideally, research using sweating thermal manikins [31] might have been preferable, as these manikins allow evaluation of clothing thermal properties while reproducing human thermoregulatory functions, including skin temperature regulation and perspiration. However, as our study achieved its objectives using simple manikins, sweating thermal manikins were not employed. Second, the study measured and verified only the temperature changes of the manikins in environments with WBGT values of ≥31 °C; thus, ambient temperature and humidity were not standardized. Furthermore, multiple factors—including manikin material properties, the dynamic state of human locomotion, and environmental ventilation, such as wind speed—were not considered. Third, the study did not account for human physiological thermoregulation (e.g., changes in blood flow and sweating). Additionally, because the surface temperature of the mannequins increased with any color combination, significant differences among data were limited. Nevertheless, the objective of this study was not to compare hot and cold environments. Lastly, the baseball uniform was worn with the baseball stirrup socks exposed, as is standard in student baseball in Japan; the style without exposed stirrup socks was not examined. This study was conducted solely in the Japanese environment. Moreover, the effect of foot and legwear materials was not comparatively examined, based on previous reports that 100% cotton and 65% polyester/35% cotton fabrics of the same color have comparable sunlight absorption rates [32].

## 5. Conclusions

We experimentally clarified the effect of foot and legwear color on lower-limb temperature during baseball played in a hot summer environment with a WBGT of ≥30 °C and continuous direct sunlight. Our results showed that wearing a white shoe, a white sock, and a white baseball stirrup sock on the lower limb reduced the increase in lower-limb temperature. This study suggests that clothing selection in baseball—specifically the use of white for shoes as well as all foot and leg apparel—may play a significant role in enhancing athletic performance and mitigating the harmful effects of heat stress. Furthermore, these findings are applicable not only to baseball but also to other outdoor sports. This research provides valuable experimental insights into the impact of elevated lower-body temperatures on health and athletic performance, as well as strategies for preventing heat stress-related health issues.

## Figures and Tables

**Table 1:** Increase in the plantar surface temperature.

Location	Shoe	Sock	Temperature (°C)	*p*-Value	ES
Dirt field	White	White	21.9 ± 4.83	-	-
		Black	21.8 ± 4.29	0.989	0.012
	Black	White	23.3 ± 5.69	0.850	−0.165
		Black	22.9 ± 6.67	0.868	−0.145
Artificial turf	White	White	18.1 ± 5.04	-	-
		Black	20.3 ± 5.67	0.634	−0.355
	Black	White	21.7 ± 5.99	0.457	−0.563
		Black	21.9 ± 5.51	0.415	−0.619

The results are presented as mean ± SD (standard deviation). Control group (each location): White shoes/white socks. ES, effect size Cohen’s *d*.

**Table 2:** Increase in the shin surface temperature.

Location	Shoe	Sock	Baseball Stirrup Sock	Temperature (°C)	*p*-Value	ES
Dirt field	White	White	White	13.9 ± 1.46	-	-
			Black	17.7 ± 1.58	0.067	−2.039
		Black	White	17.1 ± 0.46	0.082	−2.360
			Black	19.1 ± 3.23	0.138	−1.685
	Black	White	White	15.0 ± 1.19	0.483	−0.633
			Black	18.9 ± 0.83	0.023	−3.394
		Black	White	17.6 ± 1.07	0.049	−2.362
			Black	19.2 ± 0.82	0.019	−3.654
Artificial turf	White	White	White	11.6 ± 3.07	-	-
			Black	14.2 ± 5.67	0.602	−0.495
		Black	White	13.1 ± 4.44	0.644	−0.346
			Black	15.9 ± 5.93	0.321	−0.789
	Black	White	White	11.4 ± 2.88	0.937	0.058
			Black	14.7 ± 2.55	0.229	−0.950
		Black	White	12.0 ± 2.55	0.876	−0.115
			Black	15.6 ± 2.34	0.128	−1.261

The results are presented as mean ± SD (standard deviation). Control group (each location): White shoes/white socks/white baseball stirrup socks. ES, effect size Cohen’s *d*.

## Data Availability

The data presented in this study are available on request from the corresponding author due to the data are part of an ongoing study.

## Abbreviations

The following abbreviations are used in this manuscript:

WBGT	Wet bulb globe temperature
